# Endometriosis of Diaphragm: A Case Report

**DOI:** 10.22074/ijfs.2018.5379

**Published:** 2018-06-20

**Authors:** Mania Kaveh, Kobra Tahermanesh, Abolfazl Mehdizadeh Kashi, Banafsheh Tajbakhsh, Ghazal Mansouri, Kambiz Sadegi

**Affiliations:** 1 Endometriosis Research Center, Iran University of Medical Science, Tehran, Iran; 2 Department of Obstetrics and Gynecology, Zabol University of Medical Science, Zabol, Iran; 3 Department of Obstetrics and Gynecology, Kerman University of Medical Science, Kerman, Iran; 4 Pain Research Center, Iran University of Medical Science, Tehran, Iran; 5 Department of Anesthesiology, Zabol University of Medical Science, Zabol, Iran

**Keywords:** Diaphragm, Endometriosis, Laparascopy, Shoulder Pain

## Abstract

Endometriosis affects about 10% of women of reproductive age. Its main feature is the presence of stroma and endometrial glands in sites other than the uterus, mainly in pelvis. Pelvic peritoneum, ovaries, uterine ligaments, bladder, intestines, andcul-de-sac are among the affected areas. Sometimes endometriosis can be found outside of the pelvis and even above abdominal cavity, like indiaphragm.Herein, we present a case of an asymptomatic diaphragmatic endometriosis that was discovered incidentally during laparoscopy of pelvic endometriosis, as well as our appropriately proposed treatment protocol.

## Introduction

Endometriosis, which is characterized by the incidence of stroma and endometrial glands outside of the uterine cavity, is common in approximately 10% of women during their child bearing age (1). Most common site of endometriosis is pelvic peritoneum has been reported in extra-pelvic locations, like upper abdominal cavity and diaphragm, as well (2). In fact, diaphragmatic endometriosis involving the full thickness of the diaphragm includes 1-1.5% of patients diagnosed with endometriosis (1). This rare condition can be asymptomatic and has been discovered accidentally. A patient with diaphragmatic endometriosis experiences the following symptoms: upper abdominal pain on the right side, pain under the lower ribs, painful breathing, and sometimes nausea or vomiting (3, 4).

Here in, we present a case of diaphragmatic endometriosis associated with pelvic endometriosis in a 20-year-old female patient with chronic pelvic pain and dysmenorrhea with a high score. In a preliminary investigation, she was diagnosed with deep pelvic endometriosis. However, during laparoscopic surgery of the entire abdominal and pelvic cavity, diaphragmatic endometriosis was discovered incidentally, which had spread through the center and right parts of diaphragm. In this case report, we introduce a rare case of diaphragmatic endometriosis along with pelvic endometriosis and discuss its symptoms and therapeutic methods.

## Case Report

In March of 2017, a 20-year-old virgin female with achronic pelvic pain was referred to our center. The patient complained of severe pelvic pain with verbal numerical rating scale (VNRS) of 9 during the menstrual cycle. This chronic pain had lasted for almost one year. The patient did not mention dyschezia, pain during or afterurination, orother symptoms associated with diaphragmatic endometriosis, such as chest pain, shoulder pain, or right upper abdominal pain. Furthermore, she had used no hormone replacement therapy.

In abdominal examination, there was fullness on the left side, while in both rectal examination and abdominal examination, there was fullness in the posterior cul-de- sac. An immobile 10-cm mass wasfelt on the left side, whereas another immobile 5-6-cm mass was on the right side that was fixed to the uterus. 

Pelvic ultrasonography results indicated a cyst with an approximate size of 12×7 cm consisting of thick contents in the left ovary with internal septae, raising suspicion regarding formation of the tubo ovarian complex in endometrial cavity. Furthermore, the ultrasound findings showed an endometrium a cyst with an approximate dimension of 4 cm on the right side with adhesion and endometrial nodule of the posterior fundus with moderate adhesion to the rectosigmoid. Therefore, magnetic resonance imaging (MRI) was performed to exclude the left mass from adenocarcinoma, while the results showed normal upper abdominal organs, including liver, spleen, pancreas, kidneys, adrenal, as well asthe lungs. In pelvic MRI findings, there was endometrium in both adnexae along with hydrosalpinx on the left side, whereas enhancement was not reported in the left adnexal masses.

In addition, the blood test showed an anti mullerian hormone (AMH) of 1.82 and CA-125 of 125.1, while other tumor markers, including risk of ovarian malignancy algorithm (ROMA) and HE4 were normal. 

During laparoscopy, we noticed extensive endometriosis that involved the anterior and posteriorcul-de-sac, both pelvic side walls, both ovaries, and sigmoid colon. The left ovary contained a cyst measured 10-12 cm with severe adhesion to the rectum, while the right ovary contained a cyst measured approximately 6 cm with moderate adhesion to the tube and the right ovary. There was also no evidence of endometriosis in ureters. Anatomy of pelvis restored, pelvic Die corrected and a 2-cm endometriotic nodule attached to the rectovaginal septum (RVS) was shaved.

On exploring the upper abdomen, 5 to 6 areas of superficial endometriosis were discovered in the, anterior and center of the right hemi-diaphragm (Figes.1, 2), but the left hemi- diaphragm was intact. The tota Redwine l surface area of the diaphragm (left side, center, and right side) was thoroughly investigated when the patient was put into reverse Trendelenburg position. The fulguration was performed using bipolar energy for endometriotic lesions of the diaphragm. The endoscopic exploration of thoracic cavity was not performed because the patient had no symptoms of shoulder or chest pain, no history of catamenial hemothorax or pneumothorax and diaphragmatic involvement was superficial.

**Fig.1:**
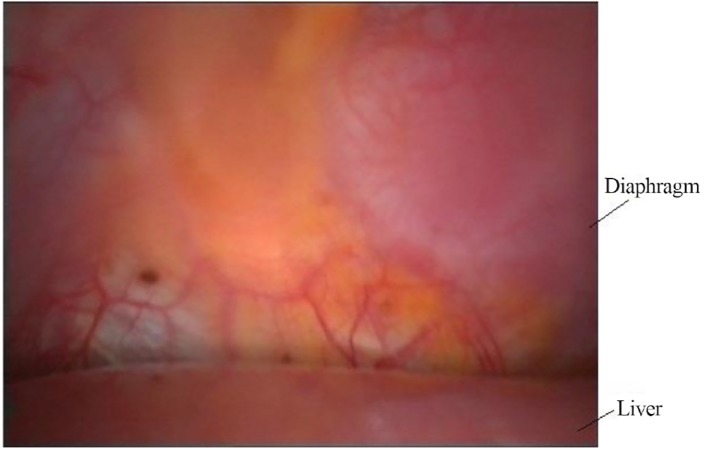
Lesions of endometriosis on the rightside and the center of the diaphragm.

**Fig.2:**
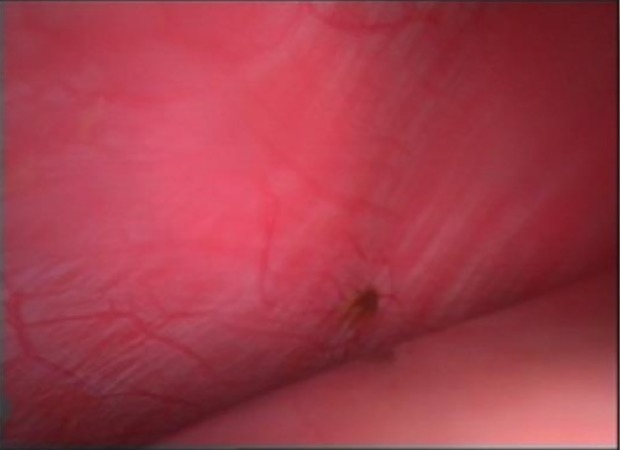
Lesion of endometriosis on the surface on right hemi diaphragm.

## Discussion

It has been reported that the incidence of endometriosis among the women of child bearing age is about 10% (5). Peritoneal cavity, especially the pelvic peritoneum, is the most common site of involvement, but endometriosis has been shown in almost all parts of the body (1). Endometriosis mainly occurs at the age of 30 to 45 years (6). The mean age for extra-pelvic endometriosis has been reported between 35 and 40 years with the prevalence of 12% (2, 3). In addition, the average age for pelvic endometriosis is 25 to 30 years (3).

Diaphragmatic endometriosis is a rare serious disorder, which has been reported for the first time as a separate term by Brews (7). Most diaphragmatic lesions occur on the right side. The pathogenesis of a higher prevalence of endometriosis in the subphrenic region is sampsons retrograde menstruation theory, which indicates that refluxed endometrium may be caught by falciform ligament in the right side of the diaphragm (1). Classical symptoms of diaphragmatic endometriosis are chest pain (pleuritic pain, especially on the right side), dyspnea, epigastric pain, shoulder pain, and upper abdomen painthat is sudden onset in many patients (2). It is noteworthy that although symptoms are usually periodic, some patients with diaphragmatic endometriosis experience continued symptoms, which are not associated with the menstrual cycle. Therefore, despite of atypical clinical symptoms, especially in patients with pelvic endometriosis, there should be a strong clinical suspicion to different types of thoracic endometriosis. 

The pain in diaphragmatic endometriosis is due to stimulation of a sensory branch of the C5 nerve root. The severity of the symptoms varies depending on the location and depth of the lesions.It has been reported that diaphragmatic endometriosis can be asymptomatic, while some women may experience no clinical symptoms or an obscure pain (8). Some serious and life-threatening conditions associated with diaphragmatic endometriosis are the results of the expansion of the fenestrations or holes in the diaphragm due to necrosis of endometriosis lesions (9). These conditions are as follows: i. Catamenial pneumothorax (CPT) is a rare condition causing the lungs to collapse during menses and responsible for about one third of spontaneous pneumothorax in women (10-12). It occurs alone or with different manifestations of thoracic endometriosis syndrome (TES), including hemopneumothorax and catamenial hemoptysis, ii. Hemopneumothorax is known as presence of blood and air in the chest cavity (13), as well as iii. Intrathoracic endometriosis nodules.

Diaphragmatic endometriosis isdiagnostic error due to the similarity of clinical symptoms with other benign or malignant disorders. About 95% of diaphragmatic lesions occur in the right side of the diaphragm, although it has been previously seen in the left side alone or both sides of the diaphragm, even in some vital structures, like the phrenic nerve. Furthermore, in most of the reported cases, the lesions occur in the anterior or posterior portion of the diaphragm and behind the liver. Therefore, due to the diversity of an organ site involvement, diaphragms and their surrounding areas should be thoroughly examined (3).

In terms of macroscopic appearance, lesions may appear in different colours and shapes that are mostly reported as bruised, purple and purple red. Computerized tomography (CT) scan or MRI may play an important role in diagnosis. Thoracic endometriosis may appear as small cystic lesions in chest radiography or CT scan (2). However, it has been shown that MRI may provide better details to diagnose endometriosis (6). In our case, MRI report showed no pulmonary endometriosis lesions.

Therapeutic measures for diaphragmatic endometriosis or suspicious thoracic endometriosis may be mainly based on the patient’s medical history. It has been strongly indicated that the best treatment choice is the expectant approach as compared to the other interventions for those patients with asymptomatic diaphragmatic endometriosis (14). 

However, for symptomatic patients, surgery will be beneficial, if the medicationis deemed to have failed (8, 15). Given a possibility of damage to the diaphragm, phrenic nerve, lungs, vessels or heart, it is crucial to choose a surgical plan after an informed consent is obtained from the patient. Also, alternative therapeutic options should be explained to the patient.

The patient’s age, the type of treatment andthe medication as well as the surgeon’s expertise should be also considered in this regard. Although there is still uncertainty about the efficiency of laparoscopic surgery in diagnosis and treatment of diaphragmatic endometriosis (3), this concern is being resolved in consultation with an expert laparoscopic surgeon regarding the use of different techniques such as proper patient positioning for an optimum view of the diaphragm and associated structures. The involvement of hidden area including the junction of the diaphragm and the posterior edge of the liver is common in the invasive conditions. In addition, the application of right sub-conundrum port or flexible laparoscope (16) may provide a precise view of the diaphragm. Simultaneous application of laparoscopy and thoracoscopic surgery (VATS) is also considered as an effective therapeutic plan in diagnosis and treatment of women with diaphragmatic endometriosis, suffering intolerable pain in the right upper abdomen and chest (due to hemopneumothorax).

The use of hormonal medications, such as danazol (oral contraceptives), has been suggested to the patients who are not interested in VATS or believe the thoracoscopy is not safe enough. The segmental resection is needed during VATS for the following disorders: tension pneumothorax, hemopneumothorax, lesions of pulmonary endometriosis, chemical pleurodesis, as well as pleurectomy (2). VATS as a procedure also provides the following abilities: resection of diaphragm implants, restoration of diaphragmatic fenestration, resection of apical blebs, and implants of lung parenchyma. This invasive procedure is known as an excisional technique dueto the complete removal of endometriosis lesions. In a number of control-randomized studies, it has been shown that complete disease eradication is the only definitive way to relieve pain, while the recurrence is negligible (17, 18). There are several different and effective methods for excision of the lesions, like vaporization, ablation, hydrodissection, and surgical scissor excision.

In our case, diaphragmatic endometriosis was discovered after inspection of the upper abdomen. In addition, the patient had nosymptoms, such as shortness of breath, shoulder pain, and right upper quadrant (RUQ) pain. Therefore, due to laparoscopic examination of the diaphragm, and appearance of lesions, the endometriosis lesions ablate. We decided to apply no other interventions for the patient.

It has been reported that the asymptomatic diaphragmatic endometriosis can be safely treated with use of laparoscopic surgery instead of laparotomy, diaphragmatic resection, or other interventions (3, 8, 9). Furthermore, VATS is known as a diagnostic and therapeutic method in selected symptomatic patients as compared to the laparotomy and thoracotomy. Medical treatment after surgery is different depending on the patient’s decision for future pregnancies. In those patients who tend to getpregnant, there is no need for further medications after surgery, but assisted reproductive technology (ART) is recommended In contrast, for those who do not want to get pregnant, suppressive hormonal treatment is used.

After discharge, considering the virginity, we prescribed suppressive hormonal medicationsfor the patient. Furthermore, we advised her to go to a hospital immediately if she experience shoulder or RUQ pain, shortness of breath and other catamenial symptoms.

## Conclusion

Endometriosis is considered as a clinical puzzle for both physicians and patients. Although many efforts have been made for both diagnosis and treatment of this disease, it is still controversial in terms of clinical symptoms, pathophysiology, disease progression as well as management. The surgeon should be fully aware of the clinical symptoms, patient’s medical history, and endometriosis lesions during a laparoscopic surgery. It is noted that if chest pain, shoulder pain, hemothorax, pneumothorax, and hemoptysis occur during the reproductive age, especially with acyclic pattern, then diaphragmatic endometriosis should be considered. Therefore, a close inspection of both anterior and posterior parts of hemi-diaphragm and applying a combined VATS/laparoscopy procedure are needed. This is especially true if there are lesions present. In order to achieve better outcomes with prevention of recurrence and re-occurring clinical symptoms, resection of any suspicious lesion is also recommended.

It is noteworthy that in contrast to conservative treatment which will be applied when endometriosis is detected during laparoscopic surgery in asymptomatic patients, however, an interventional approach is needed in symptomatic patients with post-surgical complications.

## References

[1] Nezhat C, Nicoll LM, Bhagan L, Huang JQ, Bosev D, Hajhosseini B (2009). Endometriosis of the diaphragm: four cases treated with a combination of laparoscopy and thoracoscopy. J Minim Invasive Gynecol.

[2] Honore GM (1999). Extrapelvic endometriosis. Clin Obstet Gynecol.

[3] Redwine DB (2002). Diaphragmatic endometriosis: diagnosis, surgical management, and long-term results of treatment. Fertil Steril.

[4] Funatsu K (2002). Catamenial pneumothorax: an example of porous diaphragm syndromes?. Chest.

[5] Viganò P, Parazzini F, Somigliana E, Vercellini P (2004). Endometriosis: epidemiology and aetiological factors. Best Pract Res Clin Obstet Gynaecol.

[6] Kinkel K, Frei KA, Balleyguier C, Chapron C (2006). Diagnosis of endometriosis with imaging: a review. Eur Radiol.

[7] Brews A (1954). Endometriosis including endometriosis of the diaphragm and Meigs syndrome. Proc R Soc Med.

[8] Nezhat C, Seidman DS, Nezhat F, Nezhat C (1998). Laparoscopic surgical management of diaphragmatic endometriosis. Fertil Steril.

[9] Nezhat C, King LP, Paka C, Odegaard J, Beygui R (2012). Bilateral thoracic endometriosis affecting the lung and diaphragm. JSLS.

[10] Van Mulders A, Deneffe G, Demedts M (1983). Recurring spontaneous pneumothorax in association with pleural endometriosis. Acta Clin Belg.

[11] Alifano M, Trisolini R, Cancellieri A, Regnard JF (2006). Thoracic endometriosis: current knowledge. Ann Thorac Surg.

[12] Shiraishi T (1991). Catamenial pneumothorax: report of a case and review of the Japanese and non-Japanese literature. Thorac Cardiovasc Surg.

[13] Joseph J, Sahn SA (1996). Thoracic endometriosis syndrome: new observations from an analysis of 110 cases. Am J Med.

[14] Falcone T, Lebovic DI (2011). Clinical management of endometriosis. Obstet Gynecol.

[15] Nezhat F, Nezhat C, Levy JS (1992). Laparoscopic treatment of symptomatic diaphragmatic endometriosis: a case report. Fertil Steril.

[16] Kumakiri J, Takeuchi H, Miyamoto H, Shimanuki H, Kobayashi Y, Kuroda K (2008). An advanced flexible laparoscope with wide optic angle for observing diaphragmatic lesions associated with catamenial pneumothorax. Fertil Steril.

[17] Healey M, Ang WC, Cheng C (2010). Surgical treatment of endometriosis: a prospective randomized double-blinded trial comparing excision and ablation. Fertil Steril.

[18] Wright J, Lotfallah H, Jones K, Lovell D (2005). A randomized trial of excision versus ablation for mild endometriosis. Fertil Steril.

